# Physical Activity Modulates Common Neuroplasticity Substrates in Major Depressive and Bipolar Disorder

**DOI:** 10.1155/2017/7014146

**Published:** 2017-04-26

**Authors:** Cristy Phillips

**Affiliations:** Department of Physical Therapy, A-State, Jonesboro, AR, USA

## Abstract

Mood disorders (MDs) are chronic, recurrent mental diseases that affect millions of individuals worldwide. Although the biogenic amine model has provided some clinical utility, a need remains to better understand the interrelated mechanisms that contribute to neuroplasticity deficits in MDs and the means by which various therapeutics mitigate them. Of those therapeutics being investigated, physical activity (PA) has shown clear and consistent promise. Accordingly, the aims of this review are to (1) explicate key modulators, processes, and interactions that impinge upon multiple susceptibility points to effectuate neuroplasticity deficits in MDs; (2) explore the putative mechanisms by which PA mitigates these features; (3) review protocols used to induce the positive effects of PA in MDs; and (4) highlight implications for clinicians and researchers.

## 1. Introduction

Major depressive disorder (MDD) and bipolar disorder (BP) are chronic mood disorders (MDs) that adversely affect over 400 million persons worldwide [1]. Pathognomonic features of MDD include the persistence of one or more episodes of sadness or anhedonia in a two-week period, together with a range of cognitive and somatic symptoms (e.g., changes in appetite, sleep patterns, energy level, concentration, or physical activity and feelings of worthlessness and guilt) [2]. In BP, persons exhibit similar symptoms in the depressive phase but alternate to euphoric states during the manic phase—a state characterized by excessive activity and libido and grandiose thinking [2]. Recent attention has focused on the inability of extant treatment approaches to induce remission of symptoms in a significant number of affected persons [3, 4], prompting the diversification of efforts to derive more effective treatment strategies.

Fortunately, convergent evidence demonstrates that physical activity (PA) confers neuroplastic effects [5, 6] and may serve as an effective intervention for MDs [7–12]. Physical exercise is a subcategory of PA that connotes purposeful, planned, and structured endeavors undertaken to improve skill or physical fitness level [12]. PA alters the progression of MD neuropathology by optimizing the levels of neurotransmitters [13], neurotrophic factors [13, 14], beta-endorphins [15], cortisol [16, 17], and muscle-derived protein (peroxisome proliferator-activated receptor gamma coactivator 1-*α* [PGC-1*α*]) [18]. Moreover, regular PA optimizes processes involved in neurogenesis [19, 20], immune function [21, 22], stress regulation [23, 24], antioxidant defense [25, 26], circadian rhythms [27–29], epigenetic modifications [30, 31], and the maintenance of telomere length [32–35].

Via these complex and interrelated mechanisms, PA may reduce the risk for MDs [36–38], the degree of symptoms [10, 39], the incidence of relapse [40, 41], and caregiver burden [42]. This evidence, along with its relatively low-risk profile and ease of implementation [43], has led to the incorporation of PA into basic clinical management protocols for MDs [44, 45]. Because it is important that clinicians and scientists understand the means by which PA can alter pathophysiological substrates, from both a self- and patient-education perspective, the aims of this review are to (1) elucidate key substrates implicated in MD pathobiology, (2) explore the mechanisms by which PA can mitigate them, (3) examine protocols used to effectuate the positive effects of PA in MDs, and (4) highlight implications for clinicians and scientists.

## 2. The Neurobiology of Major Depressive and Bipolar Disorders

Recent decades have noted dramatic progress in the neurobiological understanding and treatment of psychiatric conditions. On the one hand, psychiatrists have refined diagnostic categories based on clinical symptoms [46, 47]. On the other hand, neuroscientists have derived evidence of transdiagnostic biomarkers across psychiatric conditions [48–50], including those that modulate neuroplasticity substrates in MDs [51–54]. Early biologic theories focused upon neurotransmitters, particularly the biogenic amines [55]. Subsequent advances in technology heralded new abilities to characterize general and distinctive cellular and molecular mechanisms, genetic contributions, and structural correlates of psychiatric disease [56, 57]. Although a complete description of these advances is beyond the scope of this article, a cursory review of MD neurobiology will be presented before focusing on neuroplasticity substrates. The reader is referred to the following excellent reviews for a more comprehensive presentation on the neurobiology of MDD [3, 58–60] and BP [61–64].

### 2.1. Major Depressive Disorder

Neuroimaging studies of depression and analysis of surgical lesions (that induce or reverse depressive symptoms) have revealed mood circuits [65, 66]. Implicated in these circuits are several brain structures and regions, including the dorsal prefrontal cortex, ventral prefrontal cortex, anterior cingulate gyrus, amygdala, hippocampus, striatum, and thalamus [67–69]. Drevets et al. have emphasized the pathophysiological processes and dysfunction of multiple pathways that adversely affect mood circuits and structures [66, 70], including those related to genetic, epigenetic, and environmental factors. Results from a twin study suggest that the heritability of MDD is 38% [71]. Preclinical studies implicating epigenetic mechanisms have shown that maternal behavior alters the function of stress-related genes [72] and that antidepressants alter the regulation of DNA [73, 74]. Other studies have shown that depletion in neurotransmitter levels contributes to depressive symptoms [55, 75]: slow-acting neurotransmitters (e.g., dopamine, serotonin, and norepinephrine) appear to interact with signaling proteins found inside the cell membrane in a way that allows the receiving cells to process signals from glutamate and *γ*-aminobutyric acid (GABA). Accordingly, therapeutic agents for MDs were derived to increase monoamine transmission acutely, either by inhibiting neuronal reuptake or by inhibiting degradation in the synaptic cleft. While this strategy has demonstrated some utility in the alleviation of symptoms, the fact that monoamine depletion fails to produce depressive symptoms in healthy individuals [76] or worsen depressive symptoms in persons with MDD [77, 78] induced a more comprehensive search for mechanisms. Subsequent work has implicated general disruption in neurogenesis [79], trophic factor level and function [80], antioxidant defense [81], hypothalamic-pituitary-adrenal (HPA) axis function [82], immune regulation [83], neuroplasticity [84], and circadian rhythms [85, 86], changes that collectively contribute to neuronal network alterations [84, 87]. Interestingly, the patterns of disruption to neurogenesis, immune system function, and antioxidant defense in MDD are similar in many respects to the patterns of disruption that are seen in BP. Also notable is evidence that has suggested a “kindling process” wherein depressive episodes are triggered more readily over time [88] and the number of prior episodes is a better predictor of future episodes than life stress is [89].

### 2.2. Bipolar Disorder

The complex pathophysiology of BP is undoubtedly mediated by genetic and epigenetic factors acting in concert with environmental stressors [90] to effectuate functional and structural abnormalities in the interconnected limbic, striatal, and fronto-cortical neurotransmitter neuronal circuits [91] and in plasticity substrates [51–54]. A robust genetic basis for BP has been derived from familial and identical twin studies: concordance rates for BP among identical twins typically range from 40 to 70%, with the estimated heritability reaching as high as 90% [92]. Notwithstanding, genome-wide studies have failed to detect single-gene contributions, supporting the premise that BP is a polygenic condition [92]. Strikingly, while some distinctions in genetic risks exist between BP and other psychiatric conditions, a high degree of genetic overlap has been reported among BP, MDD, and schizophrenia [48, 93]. Putative interactions between genes and life stresses are thought to effectuate disruptions in homeostatic and neuroplastic mechanisms [94, 95] and increase the severity of symptoms [95]. Parallel work has demonstrated disruption of glucocorticoid signaling [96], neurogenesis [97], immune-inflammatory imbalance [98], and antioxidant defense [99], changes that contribute to increased loss of volume in brain regions vital for mood regulation and cognitive function [65]. The identification and understanding of mechanistic pathways common to BP and MDD offer an opportunity to deploy novel lifestyle interventions such as PA to target these disorders in an integrated fashion.

## 3. Neuroplasticity

**Figure 1:**
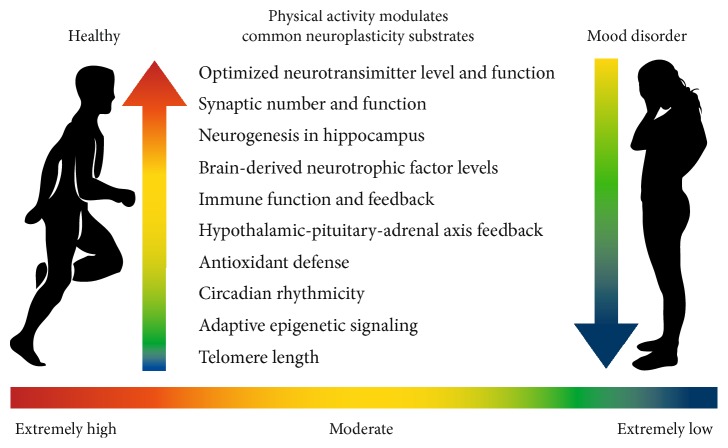
Physical activity modulates common neuroplasticity substrates in the brain. Here, the effects of various levels of PA are illustrated for the person who is healthy and the person with a MD.

The ability of the central nervous system to continuously adapt to challenges is accomplished by neuroplasticity, a process whereinneurons change and reorganize to meet the demands of the environment [100]. Neuroplasticity is dependent upon stimulus-induced synaptic activity and membrane depolarization which, in turn, induce receptor trafficking, the activation of a multiplicity of genes, and the release of neurotransmitters. The secondary messengers effectuate downstream changes in the brain that permit its resculpting. Emotional and cognitive learning, neural homeostasis, and adjustments in behavior rely on biological correlates of neural plasticity for adaptation [53, 101]. Plastic changes in the brain can be maladaptive wherein a net loss of function occurs [102], a situation that reifies in MDs [103, 104]. On the other hand, brain plasticity can be adaptive when a gain of function occurs [105]. That is, PA can modulate common neuroplasticity substrates in the brain (as described in Figure 1) and then cognitive stimulation (e.g., cognitive behavioral therapy) can increase the likelihood of behavioral change in MDs [106].

## 4. Measurement of Voluntary Physical Activity in Humans

Voluntary PA refers to locomotor activity that is not directly required for survival or motivated by an external factor (such as searching for food, shelter, or mates; interacting with competitors; or avoiding predators) [107]. Human voluntary PA occurs in a multiplicity of ways and varies tremendously in both intensity and duration, both of which modulate its physiological consequences. Several indirect and direct assessment methods have been used to investigate the effects of voluntary PA in humans—including retrospective questionnaires, surveys, activity logs, motion sensors, heart rate monitors, calorimetry, and direct observation [108]—with different methods possessing unique strengths and weaknesses. The majority of early studies used self-reports of PA, particularly given their ease of administration, cost effectiveness, positive acceptance, and lack of intrusiveness on personal habits [109, 110]. Yet, while the administration of questionnaires at the population level has proved to be a feasible method of assessment, some evidence suggests that they are the least valid and reliable measure [107, 110–112]. In contrast, direct measures of voluntary PA assess energy expenditure [113] or actual movement [114] and are less susceptible to response and recall biases [113, 115, 116]. Notwithstanding, large-scale studies using direct measures have not been feasible in the general population [117]. Additionally, it seems plausible that long-term monitoring of PA with direct measures would sacrifice face validity by increasing intrusiveness and burden on participants [107, 116]. The inverse relationship between the validity and feasibility of assessment methods [107] has prompted recent investigations to use direct measures and standardized interventions in smaller populations. Studies of this type are vital because direct measures provide a means to examine a cause and effect relationship between PA and neuroplasticity substrates. Accordingly, several investigations that aim to determine the neurobiological, psychological, and physiological effects of PA on humans are systematically reviewed in Table 1. Analysis of these investigations reveals that PA generally produces antidepressant effects and improves cognitive function, enhances quality of life, improves sleep, optimizes brain-derived neurotrophic factor (BDNF) levels and function in the hippocampus, and enhances fitness measures.

## 5. Measurement of Voluntary Physical Activity in Animal Models

Multiple animal studies have been conducted to ascertain the effects of PA on brain structure and function. Specifically, investigators have deployed a voluntary wheel-running model in rodents to simulate PA in humans [118, 119], an avenue that provides unfettered hypothesis-driven discovery. Bolstering support for the model is evidence that (1) voluntary wheel running is a self-rewarding behavior that allows rodents to choose how much to run while avoiding the stress of forced running or investigator handling [107, 120, 121], (2) rodents show a conditioned preference to the place associated with wheel running [122] and can perform an instrumental reaction to garner access [123], (3) age-related decrements in PA occur in both rodents and humans [124, 125], (4) both running wheel access in rodents and voluntary PA in humans induce changes in brain reward systems [107], and (5) voluntary wheel running and voluntary PA occur in low-energy expenditure contexts such as laboratory housing and industrialized Western society [107, 126].

The historical preference for voluntary wheel running as opposed to forced treadmill running has derived from the notion that forced running on motorized treadmills may cause the release of stress-linked hormones, which could negate neuroplastic mechanisms. Bolstering this idea are preclinical and clinical studies that have shown that restriction of control during activity is deleterious [127–129], whereas the ability to exert control is beneficial for neurobiology and mental health [130]. Recently expanding on this notion, Greenwood and colleagues [131] sought to investigate the effects of forced and voluntary PA on emotional resilience. To do so, they designed a set of experiments with five exercise conditions: sedentary controls, forced treadmill training with motion initiated by foot shock, forced running on motorized wheels designed to approximate rats' voluntary running behavior, voluntary wheel running, and voluntary wheel running in an environment matched to the forced-wheel group. Then, all animals were exposed to stress-inducing conditions (restrained in Plexiglas tubes and exposed to uncontrollable tail shock), and their escape and fear responses were monitored. Interestingly, both forced and voluntary PA on running wheels prevented the behavioral consequences of stress, but forced treadmill training with foot shocks did not. The fact that wheel runners whose activity was forcibly started and stopped in naturalistic patterns (a model that mimics circuit training) also exhibited increased stress resistance [131] suggests that the ability of PA to regulate emotion and stress resilience may be modulated by the pattern of aerobic activity as long as (1) intensity, duration, and distance thresholds are sufficient; (2) the pattern of activity is naturalistic; and (3) the degree of stress is minimized [132].

While the study of affective symptoms in rodents is difficult, well-developed behavioral assays exist, as thoughtfully characterized in a review by Holmes [133]. From these studies, it has been shown that running reverses stress-related deficits and mitigates depressive and manic behavioral outcomes. Also, most of these studies report that PA increases neurogenesis in the hippocampus, reduces the length of corticosteroid exposure following stress, improves circadian rhythmicity, increases synaptic plasticity gene activity, and increases hippocampal BDNF in models of depression. A summary of preclinical studies that investigated the effects of voluntary wheel and motorized treadmill running on neurobiological, psychological, and physiological outcomes in rodents is systematically presented in Table 2.

Evidence that impairments in neural plasticity are implicated in MDs [103] and that PA modulates common neuroplasticity substrates (neurotransmitters, synaptic number and function, neurogenesis, BDNF, inflammation, stress reactivity, antioxidant defense, circadian rhythm, epigenetic modifications, and alteration of telomere length) in MDD and BP has led to numerous attempts to harness neuroplasticity to promote healing and recovery [134], particularly as it applies to psychiatric disease [135, 136]. Fortunately, convergent evidence suggests that long-term PA is positively correlated with positive neurobiological, affective, and cognitive outcomes, as reviewed below.

## 6. Neurotransmitter Levels and Function and Physical Activity

A pathognomonic feature of MDD [55, 137] and BP [137–139] is aberrant neurotransmitter level and function, a situation that can adversely affect synaptic plasticity. It has been suggested that depressive symptoms are caused in part by a deficiency in neurotransmitter levels (serotonin, norepinephrine, and dopamine), whereas manic symptoms are caused by excess levels, a notion premised on the study of psychological and cellular actions of several psychotropic agents [140]. Both types of fluctuation are problematic given the inverted U-shaped function of neurotransmitters: moderate levels are required for optimal emotional and cognitive function [141]. Fortunately, PA can optimize the synthesis, metabolism, and release of serotonin [142–146], norepinephrine [143, 147, 148], dopamine [143, 149, 150], and glutamate [151–153].

In the serotonergic system, the ability of PA to restore depleted neurotransmitter levels in the cerebral cortex, hypothalamus, brainstem, and hippocampus [143, 154] occurs via three primary mechanisms. PA increases the relative proportion of free tryptophan peripherally [155], a condition which favors influx across the blood-brain barrier [156]. Also, PA modulates the activity of tryptophan hydroxylase [157, 158] and inactivates indoleamine 2,3-dioxygenase and tryptophan 2,3-dioxygenase, which are rate-limiting enzymes that, following stress, shunt metabolism of tryptophan towards the kynurenine pathway in lieu of the serotonin pathway [156, 159, 160].

In the noradrenergic system, PA's neuroprotective effects stem from an adaptive response [145] wherein upregulated galanin expression hyperpolarizes noradrenergic neurons and thereby inhibits excessive norepinephrine release from the locus coeruleus [161–164]. Moreover, PA increases the conversion of cortisol to its inactivated form (cortisone) [165] to dampen an individual's reactivity to stress [166–168].

In the dopaminergic system, long-term PA optimizes dopamine metabolism [149, 169] via putative mechanisms that modulate tyrosine hydroxylase activity [170], rate of dopaminergic turnover [171], and calcium levels [172, 173]. The optimization of dopamine levels is important because dopamine levels modulate motivation and reward behavior [174]. That is, deficiencies in dopamine have been related to depression [175, 176], whereas excess dopamine levels have been related to mania [98].

Recently, considerable work has focused on the putative role of glutamate and the N-methyl-D-aspartate receptor (NMDAR) in the pathophysiology of MDs. Physiologic modulation of glutamate is imperative given its central role in synaptic strength and plasticity [177–179], yet alterations in plasma, serum, and cerebrospinal fluid have been reported in MDs [180]. Notably, PA enhances glutamate turnover and prevents excitotoxicity [151, 181] by improving calcium regulation [182]. The former mechanisms reciprocally interact with corticosteroid signaling and neural plasticity processes. Conversely, PA can mitigate glutamate hypofunctioning [183]. Maddock and colleagues [152] demonstrated that long-term PA increases glutamate in the anterior cingulate cortex, a significant finding given that glutamate contributes to the production of glutathione, a pervasive antioxidant in the central nervous system. Finally, PA increases the expression of NR2A and NR2B glutamatergic receptors in the hippocampus, receptors that are associated with neurogenesis and synaptic plasticity [184, 185].

Altogether, these studies suggest that PA modulates the underlying pathobiology of MDD [186] and BP [187] by altering the levels of key neurotransmitters that regulate emotional and cognitive health. In turn, the modulation of these neurotransmitters promotes the maintenance, repair, and survival of neurons and induces changes in molecular and cellular plasticity [188–190], effects similar to those exerted by antidepressants and antipsychotics.

## 7. Synaptic Number and Function and Physical Activity

As fundamental sites of communication between a neuron and its partner cell, synapses play an important role in emotion and cognition. Synapses exhibit plasticity, wherein synaptic function and structure are modified in response to activity and factors in the cellular milieu. Long-term potentiation is one form of functional synaptic plasticity, wherein synaptic connections between synapses are strengthened following activity, a process that is fundamental to learning and memory [191]. Long-term depression is another form of functional plasticity, one that is associated with the process of forgetting, where a set of synapses display a reduced capacity to elicit a response in one another [192]. Working in concert, long-term potentiation and long-term depression regulate homeostatic plasticity and the function of neuronal circuits [193]. Structural plasticity refers to changes in the 3-dimensional structure of neurons and their connections.

Convergent evidence suggests that changes in structural and functional plasticity at the synapse are relevant to MDs [194–198] and can adversely affect emotional [199] and cognitive function [200]. Indeed, loss of synapses is a common characteristic of MDD [201, 202], resulting in disconnection and loss of function in key brain regions [80, 203, 204] such as the association cortices and hippocampal region [205]. Rodent models of BP have revealed concentrated levels of ankryin-G at the synapse (which is vital for AMPAR-mediated synaptic transmission and maintenance of spine morphology), an intriguing finding given that this gene is robustly associated with the disorder [206].

The lack of noninvasive methods for the study of synaptic function precludes direct examination in humans in vivo, prompting the use of proxy measures. Accordingly, neuroimaging studies have revealed smaller hippocampal volume in persons with MDD and BP [194–198, 207]. Moreover, the study of the hippocampus in persons with MDs has revealed that volumes are inversely associated with symptom severity and duration, but positively associated with treatment outcomes [196, 208–210].

Fascinatingly, studies have shown that PA mitigates deficits in synaptic plasticity in the hippocampus. It has been shown that for aging adults, long-term exercise counters age-related decrements in hippocampus size and protects against memory impairment [211–214]. Another study has demonstrated that healthy people who participated in long-term PA (e.g., aerobic exercise 3 times per week, 30 minutes per session, for 12 weeks) exhibited increased hippocampal volume [214], a finding that could be attributed to an increase in the number of synapses, their projections, or a combination of both. Erickson and colleagues [212] demonstrated that 1 year of aerobic exercise of moderate intensity improved memory and hippocampal volume in healthy older adults, effectively reversing the age-related loss of volume by 1-2 years. Extending these studies, Makizako and colleagues [20] demonstrated that hippocampal volume was the link between moderate PA and memory augmentation in people with mild cognitive impairment and that greater durations of moderate PA resulted in increased hippocampal volume and improved memory. Also, preclinical work has demonstrated that aerobic exercise reverses age-related decrements in long-term potentiation in the dentate gyrus region of the hippocampus [215, 216] and increases spine density in the entorhinal cortex and CA1 region [217]. Moreover, voluntary wheel running by rodents for 3 weeks changes the level of several gene transcripts known to be associated with synaptic structure and plasticity, indicating that PA elicits different gene expression profiles relevant for brain function [31]. Together, these studies suggest that PA promotes structural and functional plasticity in key regions of the brain that are adversely affected by MDs and, thereby, may be used to promote functional connectivity in persons with MDs [215, 216, 218].

## 8. Neurogenesis and Physical Activity

Neurogenesis in the adult mammalian brain is a form of experience-dependent plasticity wherein stem cells within distinct regions of the brain give rise to new neurons [219, 220] that then migrate to the dentate gyrus of the hippocampus to become integrated into circuits important for learning, memory, and emotional regulation [5, 6, 221, 222]. The 20,000,000 neurons generated over the course of a lifetime replace dead or dying neurons [219] and, in turn, enhance functional capacity to modify neural circuitry in an environmentally dependent manner [223] as both intrinsic and extrinsic factors alter the rate of neurogenesis [223, 224]. Such is relevant for persons with MDD [79] and BP [97] because the elevations in glucocorticoid levels that frequently accompany MDs reduce neurogenesis rates and effectuate volumetric decrements in the hippocampus [225]. Conversely, pharmacological blockade of glucocorticoid receptors [226] blocks decrements in hippocampal size, as can antidepressant [227] or lithium [207] administration.

Also, preclinical and clinical work suggest that rates of neurogenesis can be optimized with exercise. Voluntary wheel running in rodents potently induces neurogenesis in the dentate gyrus, changes that result from increased proliferation and differentiation of neurons [228–230]. The newly born neurons can then integrate into the hippocampal architecture, a process that takes 4–8 weeks [231]. Interestingly, the newly integrated hippocampal neurons exhibit a lower excitability threshold and enhanced neuroplastic capabilities [223]. The latter fact suggests that PA not only mitigates volumetric decrements but also may contribute to neuroplastic changes that enable the reversal of MD-related emotional and cognitive deficits. Indeed, enhanced neurogenesis in animal models has been positively correlated with improvements in learning and memory [19, 232]. A clinical study has demonstrated that regular aerobic exercise of moderate intensity (as measured by an accelerometer worn on the hip for 2 weeks) was associated with increased hippocampal volume in older adults, findings positively associated with the encoding of new memories [233]. Thus, PA-induced hippocampal neurogenesis offers considerable hope for exploiting newly born cells and their heightened plasticity to reestablish hippocampal brain circuits that have been damaged as a result of the neuroprogression of MDs [224, 228–230, 234, 235], particularly when paired with environmental enrichment (e.g., cognitive behavioral therapy).

## 9. BDNF and Physical Activity

Neurotrophins—vital proteins in the brain—contribute to the survival, growth, and maintenance of neurons [218, 236] and participate in a variety of learning and memory functions [237]. BDNF, one of the most widely distributed neurotrophins in the brain, plays a vital role in the maintenance of neurons that underlie emotion and cognition, including those adversely affected in MDs [238, 239]. The neuronal atrophy and dysfunction incurred during the course of MDs effectuate disruptions in neurotrophic support, particularly BDNF. A bevy of BDNF-related abnormalities have been associated with MDs. It has been shown that (1) serum levels of BDNF are reduced in persons with BP [240, 241] and MDD [242, 243]; (2) reductions of BDNF occur in the hippocampus in MDD and BP [244]; (3) serum levels of BDNF normalize in response to several treatments (e.g., antidepressants in MDD [242, 245], mood stabilizers and antipsychotics in BP [246–248], electroconvulsive therapy in MDD and BP [249], and PA in models of BP [250] and depression [251]); (4) polymorphisms in the *BDNF* gene are associated with MDD [252, 253] and BP [254, 255]; and (5) mature BDNF plays a critical role in brain plasticity and is intimately involved in cognitive and mood-related behaviors [236]. Accordingly, BDNF is generally regarded as a putative biomarker for BP [239, 256] and MDD [239, 257].

Recognition of the aforementioned facts, along with the fact that BDNF is highly inducible by PA, has piqued the interest of multiple laboratories in recent years [6]. Accordingly, it has been shown that PA robustly upregulates the expression of BDNF in the hippocampus of rodents [258–261], changes that endure for days [261]. Moreover, recent work has demonstrated that peroxisome proliferator-activated gamma receptor coactivator 1-alpha (PGC-1*α*) and FNDC5, muscle-derived proteins that increase following endurance exercise, regulate BDNF expression in the brain in the hippocampus of mice [262]. Such is relevant for stress-induced depression given the interaction with neuroinflammatory and neuroplasticity pathways [263, 264] via alterations in tryptophan degradation [264, 265] and 5-HT_1A_ receptor activation [266]. Thus, PA may protect against stress-induced depression by altering kynurenine metabolism [18] and, thereby, modulating BDNF levels.

Notably, PA-induced increases in BDNF have been recapitulated in unmedicated patients with MDD [267], elderly persons with remitted depression [268], and women with BP [269]. Moreover, parallel clinical studies in elderly adults have linked acute aerobic PA (until heart rate reached 85% of max capacity) with alterations in frontal cognitive functions [270], changes that may be particularly beneficial for the domains of attention, processing speed, and memory [271].

Together, these results suggest that PA may effectuate central neuroplastic adaptations via optimization of BDNF levels in persons with MDD and BP. The ability of PA to enhance BDNF release and function in the synapse, promote dendritic spine integrity, and concomitantly activate other cellular pathways [80, 179, 203, 272] is a cornerstone for neuroplastic processes that are necessary to repair and reorganize circuits damaged during the course of MDs.

## 10. Inflammation, Immune Function, and Physical Activity

The dramatic release of inflammatory cytokines following chronic mental or physical stress is a well-known harbinger of MDD [83, 273] and BP [98, 274] through mechanisms involving neuroplasticity, cell resilience, and neuronal survival [275, 276]. It has been shown that persons with MDD exhibit elevated levels of inflammatory cytokines including C-reactive protein (CRP), interleukin- (IL-) 1, IL-6, and tumor necrosis factor-*α* (TNF-*α*) [277, 278]. Yet, the most robust evidence for the role of inflammation in the causation of MDD derives from evidence that chronic cytokine immunotherapy induces depression in a significant number of patients [279, 280]. This trend is also seen in BP. Persons who were in the depressive phase exhibited increased IL-2, IL-6, IL-8, CRP, and TNF-*α* [274, 276]. Persons with BP in the manic phase exhibited increased proinflammatory markers (CRP, IL-2, IL-4, IL-6, and TNF-*α*) [274, 276], and mood symptoms have been positively correlated with IL-6 and IL-2 [276]. Fortunately, administration of cyclooxygenase-2 inhibitors and antagonists of TNF-*α* inhibits inflammatory markers in persons with MDD and BP [281–284]. Moreover, chronic lithium treatment (over 3 months) in euthymic persons with BP resulted in lower levels of peripheral blood lymphocytes secreting IL-2, IL-6, IL-10, and IFN-*γ* [285]. Another study has demonstrated that inflammatory factors were associated with cognitive performance in euthymic persons with BP. That is, TNF-*α* was associated with intrusions on the California Verbal Learning Test, IL-8 was associated with repetitions, and IFN-*γ* was negatively correlated with recollection deficits [286]. Whether the relationship of the immune response with MDD and BP is primary or secondary has yet to be determined. Nevertheless, the suggestion that PA may play an anti-inflammatory role and mitigate pathobiology in the brain warrants close consideration.

Notably, clinical studies demonstrate that PA attenuates the inflammatory process and provides a more resilient stress response [21, 22, 287, 288]. A randomized controlled trial (RCT) in healthy aging adults demonstrated that those who participated in progressive aerobic activity (15 minutes increasing to 40 minutes) twice per week for a 6-month duration exhibited a significant improvement in immune system function [287]. Similar results were found in a study of elderly women undergoing aerobic exercise (60 minutes per session, 3 times per week, for 16 weeks) [288]. Another study of fitness level (as denoted by heart rate) was associated with IL-6 and TNF-*α* response following stress [22], which is important because cytokines may adversely alter glucocorticoid receptor function to contribute to an excessive inflammatory response in MDs [289]. Finally, a study in healthy men demonstrated that PA directed monocytes towards anti-inflammatory pathways [290], a mechanism that likely supports plasticity in the brain [291]. Together, this evidence makes it seem plausible that PA induces an adaptive immune response and mitigates an exaggerated inflammatory response that can be deleterious to brain plasticity.

Admittedly, findings from human studies relating PA and immune function have varied, a situation that likely reflects unaccounted-for influences. Nevertheless, current exercise guidelines issued by the American College of Sports Medicine and the Surgeon General suggest that moderate exercise (150 minutes per week at 40% to 60% of aerobic capacity) can be deployed to induce positive immune health [292]. This notion is reaffirmed by a consensus statement drafted by international experts in the field of exercise immunology that states moderate levels of regular exercise optimize immune function [21].

## 11. HPA Function and Physical Activity

The HPA axis is an adaptive mechanism designed to respond to stress. Chronic stress is associated with hyperactivity of the HPA axis and increased levels of glucocorticoids [293, 294], even in the absence of external stressors. Several lines of evidence have implicated stress-related hyperactivity and dysregulation of the HPA axis with MDD [295, 296] and BP [62, 96]. Persons with MDD exhibit excessive HPA activity as measured by increased CRH in cerebrospinal fluid; increased cortisol in plasma, urine, and cerebrospinal fluid [297]; and increased rates of nonsuppression following administration of the synthetic glucocorticoid, dexamethasone (known as the dexamethasone suppression test) [298–300]. Persons with BP exhibited increased levels of ACTH and cortisol (basal and postdexamethasone), but not of CRH [96]. Euthymic persons with BP exhibited a flatter diurnal slope of cortisol secretion than healthy persons. Moreover, persons with a history of many episodes exhibited higher cortisol levels, reduced cortisol reactivity to daily stress, and a flatter diurnal slope than persons with fewer episodes [301]. Persons with psychotic and nonpsychotic major depression exhibited distinct patterns of HPA axis reactivity—a combination of depressive and psychotic symptoms induced a greater nadir in evening cortisol than depressive symptoms in isolation [302]. Investigation of a rodent model of BP reports stress-induced hypersecretion of glucocorticoids [303]. The relentless dysregulation of glucocorticoids in MDs can effectuate neuronal atrophy secondary to changes in neurochemistry, neuronal excitability, resilience, and plasticity of the hippocampus [196, 304–306]. In turn, these changes contribute to neurotoxicity [294] and can promote neuroprogression in MDD [307] and BP [308]. Also noteworthy is the fact that cytokines disrupt neuroendocrine function (e.g., IL-1, TNF-*α*, and interferon-*α*) by inhibiting glucocorticoid receptor signaling [275, 309] as reflected by decrements in glucocorticoid translocation and activation of glucocorticoid receptor-inducible enzymes [309, 310].

Although acute PA sharply increases levels of cortisol, regular PA mitigates an overactive stress response [23, 311]. Following stimulation, the hypothalamus secretes CRH, which then induces the release of ACTH from the pituitary gland. In turn, ACTH interacts with the adrenal gland to initiate the release of cortisol in humans and corticosterone in some animals (e.g., rodents, amphibians, reptiles, and birds) [312]. Within this context, acute exercise functions as a stressor, but regular exercise initiates neuroprotective effects. Bolstering the latter notion is evidence that long-term training reduces the response to both physical exercise [312] and other forms of stressor challenge [24], effects that may stem from altered density and efficiency of mineralocorticoid receptors, lower levels of circulating cortisol, and inhibition of cortisol synthesis [24, 312]. The ability of PA to attenuate HPA dysregulation is especially important for preventing hippocampal atrophy [313–315] and reversing cognitive deficits in aging populations [20, 212] and those with affective disorders [316], as hippocampal neurons persistently exposed to elevated glucocorticoids retract their dendrites and exhibit fewer dendritic spines [317]. Fortunately, the degree of dendritic branching in hippocampal neurons and the overall number of dendritic spines increase when animals are exposed to voluntary wheel running [217, 318, 319], alterations that enhance neuroplasticity and that mimic antidepressant actions. Translating these findings to humans at the behavioral level, it has been shown that 8 weeks of exercise improved depressive symptoms and levels of 24-hour urinary cortisol [316]. Another study demonstrated that 12 weeks of high-intensity aerobic exercise enhanced mood and optimized responsiveness of the HPA to the dexamethasone responsiveness test in persons experiencing chronic pain [320]. Altogether, these studies suggest that PA may mitigate HPA dysregulation in persons with MDD or BP, a notion that requires a further study.

## 12. Antioxidant Defense and Physical Activity

Oxidative stress is an imbalance between antioxidants and reactive oxygen species (ROS) (e.g., superoxide, hydrogen peroxide, and hydroxyl radical) [321], a problematic situation in the brain given high metabolic demands and low antioxidant capacity [322]. Oxidative stress is particularly germane to the topic of MDs given alterations in cerebral metabolic rates [323], ROS-induced lipid peroxidation, and antioxidant enzyme activity in BP [81, 324, 325] and MDD [99, 326, 327]. Moreover, oxidative changes in the milieu interfere with the stability of genomic DNA in the brain in MDs [328], changes that are correlated with severity of depressive and manic symptoms [329] and frequency of manic episodes [330].

Notably, aerobic exercise appears to increase adaptability to ROS-induced lipid peroxidation and decrease overall levels of ROS [25, 331]. These mechanisms stem in part from the ability of PA to increase antioxidant gene expression (e.g., superoxide dismutases and glutathione peroxidase) and, thereby, antioxidant enzymatic housekeeping activities in the brain [332, 333]. Together, these studies suggest that long-term exercise may optimize the enzymatic antioxidant system and mitigate oxidative damage. Such is imperative for persons with MDs given that the kinase proteins that induce structural and functional changes in synapses require specific redox environments and that synaptic activity can be modulated via ROS levels [26].

## 13. Circadian Rhythmicity and Physical Activity

Physiological processes such as feeding behavior, motor activity, hormonal secretion, and autonomic nervous function exhibit naturally occurring rhythms that are referred to as circadian rhythmicity [334]. Central to the control of circadian rhythmicity is the suprachiasmatic nucleus (SCN), a structure located in the anterior hypothalamus and composed of neurons that regulate different body functions according to rhythms that vary with the 24-hour night/day light cycle [335]. The SCN's rhythm is endogenously generated but can be synchronized to the environment (in a process referred to as entrainment) by capturing exogenous and endogenous cues that are referred to as zeitgebers. Common zeitgebers include PA, light, temperature, and food. For instance, light exposure decreases the production of melatonin (a hormone that controls sleep and wakefulness). Conversely, darkness effectuates increases in melatonin secretion, particularly two hours prior to bedtime, a change that increases the propensity for sleep [336]. Then, melatonin levels continue to increase, peak in the middle of the night, and finally decline towards the beginning of day [337]. Thereby, melatonin levels signal to the SCN the time of day. In turn, the SCN can interpret the information and use it to regulate other clocks in the brain and periphery.

Another SCN pathway involves cortisol. Cortisol levels typically peak after waking and then wane during the night, a fluctuation that can adjust the peripheral clocks in almost all organs of the body [337]. Importantly, cortisol is unable to reach the SCN. Therefore, abatement of stress provides a critical window for the SCN to resynchronize peripheral clocks [338]. Poststress resynchronization is important because desynchronization is linked to psychiatric illness [339, 340]. Indeed, reductions in nocturnal melatonin with concomitant increases in nocturnal ACTH and cortisol make it difficult to maintain sleep [341]. In turn, insufficient quantity and quality of sleep engenders disruptions in multiple regulatory systems, particularly the metabolic, immune, and cardiovascular systems [342, 343].

Multiple lines of evidence have implicated circadian disturbances and MDs. Genome-wide association studies have linked polymorphisms in core circadian genes with MDD [344, 345] and BP [345, 346]. Sleep disturbances have been documented in persons with MDD [85] and BP during the manic, depressive, and euthymic states [86, 347]. An estimated 70% of persons with MDD have reported problems with transitioning to sleep, frequent awakenings, and nonrestorative sleep [348]. For persons with BP, poor sleep quality, nighttime awakenings, and inadequate sleep are predictive factors for conversion [346]. During manic episodes, 69–99% of patients exhibited a decreased need for sleep, whereas 23–78% of persons experiencing depression exhibited hypersomnia [349]. Insomnia has been reported during the euthymic phase [349]. Some evidence suggests that persons with BP exhibit lower levels of melatonin during the euthymic, depressed, and manic phases [350]. Additionally, persons with BP exposed to a light source at night exhibit a greater suppression of melatonin synthesis than healthy controls [351], an effect that is mitigated by administration of lithium carbonate and sodium valproate [352, 353]. Similarly, a decrease in serum melatonin has been reported in persons with MDD [354]. Finally, persons with BP exhibit elevated night cortisol during the manic and depressive episodes [355, 356], an effect that can be mitigated therapeutically by administration of cortisol antagonists [357].

Given that SCN disturbances are associated with chronic stress [358] and disease [359], that persons with MDD and BP exhibit sleep disturbances and altered SCN function [360], and that gene expression patterns that regulate neuroplasticity vary with the sleep/wake cycle [361, 362], it is logical to surmise that persons with MDs exhibit circadian-related deficits in neuroplasticity [363]. Fortunately, evidence suggests that zeitgebers like PA [364], light exposure [365], social contacts, and the scheduling of rest and activity [366] can modulate rhythmic abnormalities by altering body temperature, gene expression, or the activity of several brain regions that project to the SCN (e.g., raphe nuclei and pineal gland) [367].

Supporting this notion is evidence that regular PA induces neurochemical changes that qualitatively and quantitatively improve sleep across patient populations [27–29, 368–372]. One RCT in sedentary adults with insomnia demonstrated that increasing PA to the level recommended in public health guidelines (≥150 min of moderate- to vigorous-intensity PA per week) improved sleep quality [28], whereas other studies demonstrated that adults who fail to achieve sufficient levels of PA incur sleep problems [372–375]. Other work showed that 3 months of fitness training in the middle of the day improved the consolidation of the sleep/wake cycle in older men [376]. Another study demonstrated that late-afternoon exercise improved measures of cognitive abilities in older adults [29]. Parallel investigations demonstrated that exercise induced phase delays in humans [377, 378] and accelerated re-entrainment of an acutely shifted sleep-wake cycle [379, 380]. Also, it has been demonstrated that PA between noon and evening can phase-advance melatonin rhythms [381]. In patients with nonremitted MDD, a 12-week RCT showed that PA augmentation effectuated improvements in self-reported sleep quality [382]. Later work revealed that PA-induced reductions in hypersomnia were positively correlated with reductions in BDNF and IL-1*β*, a trend that was not present in those with insomnia, suggesting differential biomarker associations for hypersomnia and insomnia [383]. Finally, preclinical work in a rodent model of BP demonstrated that both melatonin and voluntary wheel running were effective at reducing mania-related behavior [250].

While the practical implications of the aforementioned studies have yet to be clarified fully, they suggest that sleep abnormalities in MDs can be reduced with long-term PA [384–386]. Efforts to reduce sleep deficits are warranted given the morbidity associated with insomnia and the deleterious effects of sedatives. Moreover, adequate sleep is essential for plasticity processes: reactivation of regions used during the day must occur at night to consolidate memories and promote cognitive, emotional, and motor recovery [387–389].

## 14. Adaptive Epigenetic Signaling and Physical Activity

Epigenetic mechanisms have been implicated in the neurobiology of MDs [390–396]. Epigenetics refers to functional alterations in chromatin structure (e.g., DNA methylation, histone methylation, and histone acetylation) that induce modifications in gene expression, not changes in DNA sequence per se [397, 398]. That is, the chromatin structure that is determined by acetylation and methylation patterns can relax the spacing between nucleosomes to permit an increase or decrease in gene transcription, respectively [399]. Neurons utilize dynamic epigenetic modifications to regulate gene expression in an experience-dependent manner [400]. Thus, it has been suggested that neuronal plasticity is controlled in part by chromatin-remodeling processes [401], and emergent evidence has implicated chromatin modification in stress-induced illness, memory impairments, depression, and different phases of BP [402–406]. Basic support for epigenetic mechanisms in the pathobiology of MDD stems from evidence of discordance among monozygotic twins [407] along with evidence that environmental factors (e.g., early adversity) can increase the risk for depression [408, 409]. Similarly, a study of monozygotic twins discordant for BP revealed that four regions of the genome have significant alterations in methylation patterns, differences that may contribute to altered dopamine transmission and neuroendocrine function [410]. While the means by which these mechanisms impinge on MD pathobiology are not understood fully, it has become increasingly clear that these adaptations mediate long-lasting changes in vulnerability and resilience, factors relevant for MDs.

Fortunately, endogenous biochemical signals derived from peripheral cues such as PA appear to alter gene expression epigenetically [30, 411, 412] and induce physiological responses throughout the body. Thereby, PA may mitigate stress-induced changes in gene expression in persons with MDD and BP. Recent work in young and aged rats showed that two weeks of treadmill exercise induced age-dependent changes on epigenetic parameters in the hippocampus [413]. Tsankova and colleagues demonstrated that repeated stress increased histone H3K27 methylation in the hippocampus and suppressed the BDNF gene promoter region, an effect that was reversible with imipramine administration [414] or PA [415]. Recently, Januar and colleagues [416] reported similar results. They found that persons with depression at baseline, as well those with chronic late-life depression, demonstrated higher *BDNF* methylation levels, an effect that was influenced by the presence of three single-nucleotide polymorphisms (*rs6265*, *rs7103411*, and *rs908867*) [416]. Lindholm and colleagues [30] demonstrated that long-term, unilateral exercise (45 minutes, 4 times per week × 3 months) induced more than 5000 methylation changes in regulatory enhancement regions that alter gene expression, providing direct evidence that the regulation and maintenance of exercise-training adaptation is associated with epigenetic changes. In addition to preclinical data demonstrating that a multiplicity of genes that regulate synaptic structure and plasticity are altered by PA [31], the aforementioned studies make it seem plausible that epigenetic changes are responsible for a significant portion of MD-related pathobiological changes and suggest that PA is a strong candidate for reversing symptoms, particularly by promoting stable changes in BDNF gene expression. Moreover, given that chromatin modifications are associated with stress-induced illness, memory impairments, depression, and different phases of BP [393, 394, 402–406, 415, 417, 418], it seems likely that epigenetic profiles in the peripheral blood, particularly in BDNF, could be used as a biomarker for those who are susceptible to MDs, as well as for tracking therapeutic response following PA interventions in persons with MDs [30]. The latter notion is based on evidence that BDNF is robustly upregulated centrally and peripherally [419–422] following acute and long-term exercise [423, 424] and that plasma BDNF levels are linked to alterations in brain BDNF levels [270, 425], synaptic plasticity, and learning ability [271].

## 15. Telomere Length and Physical Activity

Recent evidence suggests a correlation between telomere length and MD pathobiology. Telomeres—the tiny, protective caps found on the terminals of DNA strands—protect DNA from damage during the processes of cell division and replication. Telomeres naturally shorten and fray with cellular aging, a hallmark process that is accelerated by adverse health and lifestyle circumstances [426]. Although the specific mechanisms underlying telomere shortening remain unknown, recent evidence has intimated that telomere shortening in MDs may be attributed to oxidative stress and inflammation, processes that can induce telomeric DNA damage and accelerate aging by 10 years [99, 427–429]. Moreover, it has been shown that telomere maintenance is critical for stem cell function in the context of persistent neuronal turnover and that adult stem cells exhibit high levels of telomerase (an enzyme that extends the telomere sequences after cell division) [430–432], making it seem likely that hippocampal neurogenesis depends on telomere dynamics [433].

Shorter telomere length has been reported in persons with remitted MDD and current MDD, findings that correlate with the severity and duration of symptoms [434] andmayrelatetoaperson'soverall state of resiliency [435]. In postmortem samples, shortened telomere length was observed across brain regions, including the hippocampus in persons with MDD [436]. Also, telomere shortening has been reported in persons with BP [437], and the length was inversely correlated with the number of depressive episodes and response to lithium [438]. Strikingly, recent findings show that lithium increases the expression of the gene that encodes telomerase in human neural progenitor cells [439], intimating that the accelerated aging processes that occur in BP may be mitigated through the neuroprotective effects of lithium.

Similarly, recent clinical studies suggest that PA has protective effects on telomeres [32]. In general, persons who report more PA have significantly longer telomere length [440, 441]. Loprinzi and colleagues [33] reported a clear dose-dependent relationship between the degree of PA engagement and shorter leukocyte telomere lengths: persons who participated in a single type of PA were 3% less likely to have very short telomeres compared to a sedentary individual, whereas persons who reported that they engaged in 4 types of PA were 59% less likely to have very short telomeres. Werner and colleagues [442] reported that peripheral blood leukocytes from professional endurance athletes exhibited increased telomerase activity and reduced expression of cell-cycle inhibitors compared with those from untrained individuals. Preclinical evidence suggests that PA has a protective effect on telomere length in neurons in brain regions associated with depression [34, 35].

Together, this emerging evidence suggests that PA may attenuate MD-related disease and provide a means to protect telomeres from accelerated aging. The fact that telomere maintenance is critical for stem cell function in the context of persistent turnover [430–432] makes it seem likely that hippocampal neurogenesis depends on telomere dynamics and that PA may positively affect these processes, a notion that awaits further investigation.

## 16. Implications for Translation, Unresolved Issues, and Future Directions

Finding an effective treatment for MD-related impairments and symptoms remains an unmet goal. Notwithstanding, the use of animal models and human research have resulted in considerable progress toward understanding common neuroplasticity substrates that are implicated in MDD and BP, along with the identification of new targets and biomarkers for therapeutic intervention. Presented here is a broad assessment of biomedical evidence intimating that PA can optimize neuroplasticity substrates and processes across brain regions so that neuronal networks responsible for emotional and cognitive regulation can reestablish their connectivity to better meet environmental and biological demands in a use-dependent manner [84, 399, 443–445]. Consistent with the delayed effects of PA, this process would take weeks to induce network changes of sufficient magnitude to effectuate downstream changes in mood and behavior. Another key thrust of this notion is that the interplay between these neuroplasticity substrates is important for appropriate network function, not isolated factors per se. Studies of these interrelated processes are imperative given evidence that interventions applied earlier in the course of disease are more likely to achieve disease modification, whereas those applied later may have a significant but more limited effect [446, 447].

While the beneficial nature of PA for persons with MDD has been clearly established [10, 448], there is a relative dearth of evidence for persons with BP. Cooney and colleagues conducted a meta-analysis of high-quality RCTs published up to March 2013 to determine whether there is enough evidence to support the deployment of PA in clinical populations with depression [10]. Thirty-nine studies with a total of 2326 participants were included in the review. The authors reported that exercise produced effects comparable to treatment with antidepressants or psychotherapy. Another meta-analytic study reported that aerobic exercise moderately reduced the signs of depression, with populations over 60 years of age deriving the greatest effect [448]. A more recent meta-analytic review attempted to determine optimal parameters for using exercise to treat depression (e.g., frequency, intensity, duration, and type of exercise). They noted that all five RCTs meeting inclusion criteria were aerobic in nature (walking on treadmill or outdoors, cycling on a stationary bike, or training on an elliptical machine) [449]. Moreover, positive evidence was found that aerobic exercise of moderate intensity, undertaken 3 times weekly for a minimum of 9 weeks, was successful in treating depression [449]. Separate clinical studies in persons with BP suggest that exercise, in combination with mood stabilizers, improves outcomes [450, 451], and routine exercise is a common wellness strategy practiced by persons with high-functioning BP [452]. For instance, one preliminary trial noted a trend towards significance for persons with BP in an inpatient psychiatric treatment unit that participated voluntarily in a walking group during their admission [451]. Another preliminary trial in persons with BP found the addition of 100 minutes of weekly exercise to baseline activities, in conjunction with nutrition and wellness activities, effectuated improvements in quality of life, depressive symptoms, and weight loss [453]. A meta-analysis of six studies reported the feasibility and benefit of PA in persons with BP, particularly noting decrements in symptoms of depression, anxiety, and stress [454].

Given that it is generally held that positive, supportive relationships have a beneficial effect on the maintenance of psychological health [455], further consideration of the influence of social interactions during group exercise is warranted, particularly in aging individuals. Group activities such as exercise may help retirees deal more effectively with the loss of social relationships and influence in the workplace by providing an opportunity to meet and socialize with others [456]; disproving stereotypes; and promoting feelings of autonomy, relatedness to others, and competence [457]. In turn, positive social relationships may facilitate the adherence to leisure-time PA. A large prospective cohort study of middle-aged adults found that adults who experienced high levels of emotional support were more likely to adhere to recommended levels of leisure-time PA at follow-up in comparison to those with lower levels of social support [458]. Alternatively, social participation and engagement may reflect an index of behavioral plasticity [459, 460] wherein social engagement helps aging persons to compensate for changes in neural systems involved in emotional and cognitive functioning, a notion that awaits future investigation.

Clearly, large-scale, multiple-site clinical investigations that study the relationship between exercise and MDs are needed. The degree to which polymorphisms (common variations in gene sequence) determine an individual's response to PA is largely unknown. Future work that combines the study of genetic background (e.g., polymorphisms in BDNF), postexercise serum BDNF, and affective and cognitive measures with neuroimaging studies of MD-related circuits could be used to determine the “dose” of PA requisite to mitigate structural and functional changes in the brain across patient populations. Moreover, strategies for overcoming the core symptoms of depression (e.g., loss of interest, motivation, and energy; low self-worth feelings and self-confidence; psychosomatic complaints; and comorbid health problems) and mania need to be clearly delineated and articulated so that exercise can be personalized.

In summary, the data presented here suggest that moderate PA—a target that is practical, well tolerated, and likely to optimize exercise adherence—can be used to improve the neurobiological impairments and behavioral symptoms associated with MDD and BP. Because of this, PA should be advocated to reduce symptoms, prevent relapse, and mitigate residual symptoms vis-a-vis the promotion of good health habits [461]. The success of prevention campaigns will require significant changes in philosophy and approach. Evidence suggests that 50% or less of mental health professionals recommended exercise for depression, and less than a third of those felt confident in making individualized recommendations [364, 462]. Ultimately, it is hoped that a more consolidated treatment view and research approach will translate into novel therapeutic avenues of preventive and curative value for persons with MDD and BP.

## Figures and Tables

**Table 1:** Clinical trials of physical activity in persons with mood disorders. To determine the effects of PA on the brain in humans affected by MDs, a computer search of MEDLINE using the terms “mood disorder,” “physical activity,” and “exercise” was used to produce a list of interventional studies. Then, manual searches of key references were performed to identify additional studies. Articles met inclusion criteria if they were peer-reviewed interventional studies in persons diagnosed with MDD or BP. Articles were excluded if they were reviews, case reports, conference abstracts, expert opinions, or clinical studies of adolescents. Duplicate articles and those not available in English language were excluded also. Based on this search and subsequent screening, 37 articles that spanned from 1987 to 2016 were identified. Whereas extensive variations existed in the studies with regard to age, sex, degree of symptoms, phase of disease, and setting, 97% of RCTs (31 out of 32) that measured behavioral outcomes reported positive associations between PA and recovery from depressive symptoms [40, 41, 267, 316, 463–489] by utilizing training ranges of 100–250 min per week for a duration of 2–6 months [40, 41, 316, 463–468, 472–474, 476–485, 487, 488]. One report achieved relief of depressive symptoms following 60 minutes of PA for a duration of 5 weeks [489], whereas another study reported that participants obtained relief following PA 30 min/day for a duration of 1 week [470]. The modalities used in the programs varied, but most of the programs deployed some form of aerobic activity as a core component [40, 41, 96, 464–483, 485–488, 490, 491]. Notably, the one study that failed to find an association between PA and depressive symptoms used a relaxation group as a control [492], a fact that may be problematic given preliminary evidence that stress reduction activities reduce cortisol abnormalities and, in turn, may mitigate depressive symptoms [489]. The remaining studies reported that PA reduced sleep problems [382, 383, 487]; normalized BDNF levels in some studies [267, 268], but failed to do so in others [490]; and reduced cortisol levels [489]. Nevertheless, extant RCTs are still few and leave many questions unresolved.

References	Sample	Modality	Frequency & duration of PA	Assessment
[463]	Mean age of 75 y/o with MDD (*n* = 121)	Sertraline only; sertraline + supervised nonprogressive PA (<70% peak heart rate); sertraline + supervised progressive aerobic activity (60% peak heart rate)	60 min/session 3 d/wk for 24 wks	Reduced depressive symptoms on HAM-D and CGI in all groups, but earlier and higher remission rates in exercise groups at 4, 8, and 12 wks
[464]	50 y/o or greater with MDD (*n* = 156)	Aerobic exercise (70–85% max HR); aerobic exercise (70–85% max HR) + standard medication; or standard medication only	Supervised 45 min sessions 3 d/wk × 16 wks	Reduced depressive symptoms on BDI and HAM-D in all groups, but response was quicker in medication-only group
[465]	19–78 y/o with depressive symptoms (*n* = 112)	Aerobic exercise outside during daylight hours (60% max HR) + prompts to take a specific vitamin regimen or control	20 min per session 5 d/wk × 8 wks	Reduced depressive symptoms in both groups, but more so in exercise group; specifically, ↓ depressive symptoms on CES-D in exercise group; ↓ anger and tension on POMS in exercise group; ↑ vitality in exercise group
[466]	18–65 y/o with MDD (*n* = 62)	Add-on aerobic exercise × 10 wks; add-on basic body awareness therapy × 10 wks; or single consult for advice on PA + care as usual	55–60 min session 2 d/wk × 10 wks; group basic body awareness therapy 2 d/wk × 60 min; or advice on PA on one occasion	Reduced depressive symptoms on MADRS in all groups (−10.3 in aerobic PA, −5.8 in body awareness, and −4.6 in advice only group); ↑ cardiovascular fitness gains in aerobic exercise group; ↓ self-rated depression symptoms in PA and basic body awareness groups
[41]	50 y/o or greater with MDD (*n* = 133)	Aerobic activity (70–85% max HR); aerobic activity (70–85% max HR) + sertraline; or sertraline only	Supervised 45 min sessions 3 d/wk × 16 wks then follow-up 24 wks after study conclusion	Reduced depressive symptoms on HAM-D; ↑ rate of partial or full recovery from depressive symptoms on HAM-D in exercise group; and ↓ rate of relapse for MDD in exercise group
[316]	18–20 y/o with mild to moderate depression (*n* = 28)	Exercise regimen or usual daily activities	50 min sessions 5 d/wk × 8 weeks for each regimen	Exercise regimen reduced depressive symptoms on CES-D; ↓ cortisol; and ↓ urinary secretion of epinephrine
[467]	20–64 y/o with MDD (*n* = 82)	Aerobic exercise + care as usual or care as usual only	Progressive exercise 45–60 min per session 3 d/wk × 8 wks	Combination of exercise + fluoxetine group exhibited greater reduction in depressive symptoms on BDI and ICD-10 than fluoxetine alone
[468]	18–35 y/o with MDD or minor depression (*n* = 40)	Aerobic (80% max HR); strength training (50–60% max HR); or control	Supervised sessions 4 d/wk × 8 wks	Reduced depressive symptoms on BDI and HAM-D in both exercise groups following intervention and at 12 mo follow-up
[469]	20–45 y/o with diagnosis of MDD (*n* = 80)	4 aerobic exercise treatment groups that varied according to intensity: low dose (7.5 kcal/kg/wk for 3 or 5 d/wk × 12 wks); high dose (17.5 kcal/kg/wk for 3 or 5 d/wk × 12 wks); or control	Supervised aerobic activity × 12 wks	Reduced depressive symptoms on HAM-D for high-dose aerobic exercise (17.5 kcal/kg/wk 3–5 d/wk)
[470]	20–53 y/o with MDD (*n* = 38), somatization syndrome (*n* = 26), or healthy controls (*n* = 47)	Aerobic exercise or control	30 min/d for 1 wk or reduced PA for 1 wk	Reduced depressive symptoms on BDI 2 following 1 wk of exercise in persons with MDD, but not other groups; ↑ monocytes in healthy controls, but not in persons with MDD or somatization syndrome
[471]	18–65 y/o with MDD and sedentary lifestyle and with residual cognitive or attention impairments following tx with SSRIs for 8–12 wks (*n* = 39)	High-dose aerobic exercise (target of either 16 KKW—the equivalent to walking 4 mph × 210 min/wk) or low-dose aerobic control (4 KKW—the equivalent to walking 3.0 mph for 75 min/wk)	Initial supervision during sessions then transition to home-based program × 12 wks	Reduced depressive symptoms in both groups on IDS-C, but greater effect in high-dose exercise group; high dose PA ↑ spatial working memory and both groups ↑ cognitive function (psychomotor speed and executive function)
[472]	60 y/o or greater women who were overweight or moderately depressed (*n* = 106)	Add-on supervised aerobic exercise + strengthening activities or usual care	Supervised 50 min session 3 d/wk × 24 wks	Reduced depressive symptoms and anxiety on GDS, STAI, and EQ-5D in intervention group; ↓ BMI in intervention group
[473]	40 y/o or greater with diagnosis of MDD (*n* = 102)	Supervised aerobic exercises (70–85% of max HR); sertraline; or placebo	45 min session 3 d/wk × 16 wks	Reduced depressive symptoms in both groups on HAM-D and BDI along with higher remission rates compared to placebo; ↔ between groups in verbal memory, verbal fluency, or working memory
[40]	Mean age of 51 y/o with MDD and sedentary (*n* = 202)	Supervised aerobic exercise (70–80% of max HR); home-based exercise; sertraline; or placebo	45 min session 3 d/wk × 16 wks	At 12 mo follow-up, exercisers who reported 180 min/wk exhibited reduced depressive symptoms on HAM-D scores and a ↓ risk for relapse in comparison with persons who reported 0 min of exercise
[474]	18 y/o or greater with MDD (*n* = 42)	Structured group exercise (50% max HR) or usual care	45 min session 3 d/wk × 6 wks	Reduced depressive symptoms on MADRS and BDI-2 in both groups, but ↑ response (> 50% decrease of symptoms on MADRS) in exercise group; ↓ diastolic blood pressure in exercise group; ↓ waist circumference in exercise group; ↑ HDL in exercise group; ↑ cardiorespiratory capacity in exercise group
[475]	75 y/o or greater with depressive symptoms (*n* = 193)	Individualized; home-based exercise program (i.e., balance, strength, and aerobic activity); or control	52 wks	Reduced depressive symptoms on GDS and ↑ mental health-related quality of life in both groups, but no difference between groups
[476]	18 y/o or greater with depressive symptoms (*n* = 23)	Low-frequency aerobic exercise (within target HR); high-frequency aerobic exercise; or high-frequency aerobic exercise + group team building intervention	1 aerobic activity 30 min session 1 d/wk × 8 wks; 30 min session 3–5 d/wk × 8 wks; 30 min session 3–5 d/wk + group team building × 8 wks	Persons in high-frequency aerobic groups exhibited reduced depressive symptoms on BDI-2, but team-building intervention ↔ depressive symptoms
[477]	22–63 y/o with depressive symptoms (*n* = 80)	Aerobics + bright light or aerobics + normal light	Individualized aerobic training 2-3 d/wk × 8 wks	At 8 wks, reduced depressive symptoms on HAM-D and ATYP in both groups, but greater effect in aerobics + bright light group; ↑ in vitality on RAND in both groups, but more so in bright light group
[478]	26–63 y/o with depressive symptoms (*n* = 98)	Aerobics + bright light; aerobics + normal light; or stretching in bright light	Supervised sessions 2 d/wk × 8 wks	Reduced depressive symptoms on HAM-D in both aerobic groups; reduced depressive symptoms on SIGH-SAD-SR in aerobic + bright light group; ↔ in serum lipid levels or BMI in any group
[485]	31–52 y/o with dysthymia and MDD (*n* = 99)	Add-on aerobic exercise (70% max HR); nonaerobic exercise; or usual care	Supervised 60 min sessions 3 d/wk × 8 wks	Reduced depressive symptoms on BDI in both exercise groups; ↑ VO_2_ max in aerobic exercise group
[479]	21–70 y/o or greater with MDD or BD (*n* = 75)	Chronotherapeutic intervention (consisting of wake therapy, bright light therapy, sleep phase advance, and sleep time stabilization) or individualized aerobic exercise plan	30 min sessions 5 d/wk × 29 wks	Reduced depressive symptoms on HAM-D in both groups, but even greater response in chronotherapy group—at 9 wks remission rate was 45% for chronotherapy group versus 23% for PA group and at 29 wks remission was 62% for chronotherapy group versus 38% for PA group
[480]	53 y/o or greater with mood disorder who were poor responders to antidepressant meds (*n* = 86)	Add-on exercise (aerobic, strengthening, and stretching) or health education talks	Supervised activity for 60 min session 2 d/wk × 10 wks	Reduced depressive symptoms on HAM-D in both groups, but response more positive in exercise group
[487]	65 y/o or greater with and without depressive symptoms who are sedentary (*n* = 451)	Aerobic exercise (60 to 80% max HR) or progressive strength training (50–75% 1 rep max)	Supervised training 60 min session 3 d/wk × 10/wks	Reduced depressive symptoms on GDS in both strength training and aerobic exercise groups; ↑ plasma BDNF in strength training group
[481]	60 y/o or greater with osteoarthritis of knee and depressive symptoms (*n* = 438)	Aerobic exercise (50–70% max HR); strength training; or health education	Supervised walking 60 min session 3 d/wk then home-based aerobic activity × 15 mo or supervised progressive strength training 60 min session 3 d/wk × 3 mo + home-based continuation of training × 15 mo	Reduced depressive symptoms on CES-D in aerobic exercise group; ↔ depressive symptoms on CES-D in strength training group; both aerobic and strength training ↓ pain, ↓ self-reported disability, and ↑ walking speed
[486]	50 y/o or greater with MDD (*n* = 200)	Add-on aerobic home-based program (target of 150 min per wk) and strength training + usual care or usual care only	Exercise 3 d/wk for strength training for all major muscle groups + 30 min session aerobic activity 5 d/wk × 12 wks	Reduced depressive symptoms on MADRS in both groups at 12-, 26-, and 52-week follow-up assessments
[489]	18–65 y/o with MDD (*n* = 60)	Add-on yoga to quetiapine fumarate or escitalopram or no yoga	Supervised 60 session 1 d/wk × 5 wks	Reduced depressive symptoms on HAM-D; trend towards ↓ cortisol secretion in both groups
[482]	18–60 y/o with MDD (*n* = 26)	Add-on aerobic exercise at patient selected intensity + usual care or usual care only	16.5 kcal/kg/wk × 3 d/wk	Reduced depressive symptoms on HAM-D and QoL measure in psychological domain
[483]	18–60 y/o severely depressed inpatients with MDD (*n* = 50)	Add-on aerobic PA (with goal of 15.5 kcal/kg/wk) + usual care or usual care only	Supervised session 3 d/wk (mean length 23.36 days ± 9 days)	Reduced depressive symptoms on HAM-D and ↑ quality of life (World Health Organization Quality of Life Assessment Instrument-Brief version (WHOQOL-BREF) during second wk of treatment and at discharge
[484]	69–73 y/o with MDD, minor depressive symptoms, or dysthymia (*n* = 32)	Progressive resistance training (3 sets of 8 repetitions of 80% 1 rep max) × 10 wks + unsupervised exercise or health education	Supervised 45 min sessions 3 d/wk × 10 wks followed by unsupervised resistance training 2-3 d/wk × 10 wks	Reduced depressive symptoms in exercise group on BDI at 20 wks and 26 mo follow-up; ↑ morale on measures of aging on the Philadelphia Geriatric Morale Scale
[488]	18–55 y/o with MDD (*n* = 57)	Add-on aerobic exercise (60–85% VO_2_ max) + sertraline or sertraline only	Supervised sessions 4 d/wk × 4 wks	Reduced depressive symptoms on HAM-D in both groups, but response occurred with lower dosage in exercisers; ↑ VO_2_ max in exercisers
[492]	18–55 y/o with MDD who were medicated and unmedicated and received psychotherapy (*n* = 165)	Strength training (2 or 3 trials of 12 reps at 50% max and increasing to 8 reps of 75% max); aerobic exercise (70% max heart rate); or control (stretching and relaxation groups (*n* = 55 for each)	Supervised training 90 min per session 2 d/wk × 16 wks	↔ in depressive symptoms between three groups on HAM-D at 4 mo and 12 mo; ↔ in cognitive symptoms between the three groups at 4 mo and 12 mo
[268]	50 y/o or greater with remitted MDD (*n* = 35)	Modified incremental walking protocol	Supervised single 30 min exercise bout	↑ BDNF towards levels comparable to healthy controls
[267]	22 y/o or greater with MDD (*n* = 18)	Progressive exercise until 125 beats per minute	Supervised single aerobic exercise bout	↑ BDNF
[383]	18–70 y/o with nonremitted MDD (*n* = 126)	Augmentation of SSRI with 16 kilocalories per kilogram of body weight per wk × 12 wks (equivalent to 150 min per wk at moderate intensity) or 4 kilocalories per kilogram of body weight per wk × 12 wks	Sensor monitored and partially supervised × 12 wks	↓ in hypersomnia on IDS-C, a change that was correlated with ↓ BDNF and ↓ IL-1*β*; lower baseline levels of IL-1*β* predicted greater improvements in insomnia
[490]	18–60 y/o with MDD (*n* = 79)	Aerobic exercise (80% aerobic capacity) or control	Supervised 45 min sessions 3 d/wk × 3 mo	↔ hippocampal volume; BDNF; VEGF; or IGF-1 in exercise group
[382]	18–70 y/o with nonremitted MDD (*n* = 122)	Augmentation of SSRI with 16 kilocalories per kilogram of body weight per wk × 12 wks (equivalent to 150 min per wk at moderate intensity) or 4 kilocalories per kilogram of body weight per wk × 12 wks	Sensor monitored and partially supervised × 12 wks	↓ insomnia as measured on IDS-C in both groups
[491]	18–60 y/o with MDD (*n* = 53) versus healthy controls (*n* = 58)	Aerobic exercise (80% max heart rate) or control	Supervised sessions 45 min session 3 d/wk × 3 mo	↓ at-rest levels of copeptin in participants with high exercise compliance

ATYP: Atypical Depression Symptoms Addendum to Hamilton Depression Rating Scale; BAI: Beck Anxiety Inventory; BDI: Beck Depression Inventory; CES-D: Center for Epidemiologic Studies Depression; GWB: General Well-Being Schedule; GDS: Geriatric Depression Scale; HAM-D: Hamilton Depression Rating Scale; ICD-10-D: International Classification of Diseases-Depression; IDS-SR: Inventory of Depressive Symptomatology-Self Reported; POMS: Profile of Mood States; GCPS: Graded Chronic Pain Scale; CGI: Global Improvement of Depression; MADRS: Montgomery and Asberg Depression Rating Scale; QoL: quality of life; QALY: quality-adjusted life years using EuroQol (EQ-5D); RAND: RAND 36-Item Health Survey; SIGH-SAD-SR: Seasonal Affective Disorders Version Self-Rating Format; STAI: State-Trait Anxiety Inventory; WHOQOL-BREF: World Health Organization Quality of Life Assessment Instrument-Brief version.

**Table 2:** Preclinical studies of physical activity in rodent models of mood disorder. To determine the effects of PA on the brain of rodents, a computer search of MEDLINE using the terms “voluntary wheel running,” “rodents,” “depression,” and “mania” was used to produce a list of studies. Then, manual searches of key references were performed to identify additional studies. Articles met inclusion criteria if they were peer-reviewed and performed in rodent models of depression or bipolar disorder. Articles were excluded if they were reviews, conference abstracts, or expert opinions. Duplicate articles and those not available in English language were excluded also. Based on this search and subsequent screening, a total of 28 articles that spanned from 2001 to 2016 were identified. Of those studies, 20 examined the effects of PA on behavioral outcomes. Strikingly, PA mitigated adverse outcomes in 95% (19 out of 20) of the studies that utilized behavioral measures, including those that examined depressive [493–508], anxiety [493, 496], and social-[495] and fear-avoidant behaviors [131, 509]. Another study reported that exercise mitigated manic-like behavior [250]. The remaining studies generally reported that exercise mitigated cognitive impairments [131, 499, 509], optimized the glucocorticoid response [500, 501, 509, 510] and neurotransmitter levels [497, 498, 511], optimized BDNF in the hippocampus [251, 493, 499, 500, 504, 505, 512, 513], increased neurogenesis [251, 502, 505, 506, 513], enhanced sleep [250, 514], and increased synaptic markers [508].

References	MD model	Age (wks)	Treatment modality	Duration (wks)	Measure	Outcome
[496]	Wistar rats exposed to chronic unpredictable stress	8	Voluntary wheel running	4	Sucrose preference, elevated plus-maze, elevated T-maze, and forced-swim test	Reduced depressive-like symptoms and ↓ anxiety symptoms in stressed rats
[499]	C57BL/6 mice exposed to chronic uncontrollable stress	8-9	Voluntary wheel running	4	Forced swim, tail suspension, water maze, and BDNF	Reduced depressive-like symptoms, ↑ spatial memory, ↑ mature BDNF
[493]	C57BL/6 mice exposed to chronic uncontrollable stress	“Adult” status, but actual age not specified	Voluntary wheel running	3-4	Learned helplessness, forced swim, tail suspension, elevated plus maze, and BDNF	Reduced depressive-like symptoms and ↓ anxiety symptoms in wheel runners, ↑ hippocampal BDNF mRNA
[500]	Sprague-Dawley rats exposed to chronic uncontrollable stress	Not specified	Voluntary wheel running	4 wks	Sucrose preference test, open field test, Morris water maze, corticoste rone, BDNF RNA, and glucocorticoid receptor RNA	Reduced depressive-like symptoms, ↑ hippocampal BDNF RNA following stress, ↓ corticosterone following stress, ↑ glucocorticoid RNA receptor following stress
[501]	Sprague-Dawley rats exposed to chronic uncontrollable stress	“Adult” status, but actual age not specified	Voluntary wheel running	4 wks voluntary wheel running prior to stress followed by 4 wks voluntary wheel running after stress	IgM antibodies, IgG2a antibodies, and distance ran	Voluntary wheel running reduced stress-induced depressive-like symptoms and ↓ stress- induced alterations in immune function
[503]	Fischer F344 rats exposed to chronic uncontrollable stress	“Adult” status, but actual age not specified	Voluntary wheel running	2 and 6	Freezing behavior and shuttle-box escape learning	6 wks running after exposure to uncontrollable stress reduced depressive-like symptoms and anxiety symptoms in runners
[515]	Fischer F344 rats exposed to chronic uncontrollable stress	8	Voluntary wheel running	5	Social exploration, shock-elicited freezing, escape behavior, corticosterone, and body weight	Runners exhibited ↓ body weight, ↓ anxiety and reduced depressive-like symptoms, and ↓ corticosterone response to stress
[505]	C57BL/6 mice exposed to chronic uncontrollable stress	8	Stressed animals with no treatment, a dietary supplement (antioxidants), voluntary wheel running, or both dietary supplement & free access to running wheel	4	Neurogenesis, hippocampal BDNF mRNA, VGF serum, and saccharin preference	Combination of diet and exercise (but neither alone) reduced depressive-like symptoms, ↑ neurogenesis in dentate gyrus in diet + exercise group, ↑ BDNF mRNA in hippocampus of exercise + diet group, and trend towards ↑ VGF in exercise + dietary supplement group only
[506]	ICR mice exposed to chronic uncontrollable stress	8	Treadmill running 60 min, treadmill running + SU1498 [a VGF receptor (Flk-1) inhibitor], and control	2	Open-field test, forced swim test, open-field test, BrdU, Ki67, and CD31 immunohistochemistry	Treadmill group exhibited ↑ hippocampal neurogenesis in dentate gyrus and ↓ anxiety and reduced depressive-like symptoms in a manner that is dependent on VEGF-Flk-1 signaling
[498]	Swiss mice	Age not specified	Voluntary wheel running	3	Forced swim, tail suspension, and open-field test	Reduced depressive-like symptoms, an effect that appeared related to availability of bioamines
[507]	Diurnal sand rats housed in either short photoperiod (SP) (5 hr light/19 hr dark) or neutral light (12 light/12 dark)	24	Voluntary wheel running	3	Elevated plus-maze, forced-swim test, and social interaction	↓ disruptions in activity rhythms of SP/exercise animals, ↓ anxiety and reduced depressive- like symptoms for SP/running wheel group, ↑ number and duration of social interaction for SP/running wheel group
[504]	C57BL/6J mice	6-7	Voluntary wheel running	4	VGF protein, BDNF protein, plasticity genes (Egr2, Grb2, ornithine decarboxylase-1, synapsin-1, and synCAM), forced-swim, and tail-suspension tests	Reduced depressive- like symptoms, ↑ in VGF protein and ↑ in BDNF protein in hippocampus, altered synaptic plasticity gene profile activity
[502]	Flinders Sensitive Line (FSL) rat	22	Administered escitalopram, escitalopram + voluntary wheel running, vehicle diet, or vehicle diet + voluntary wheel running	4	BrdU immunohistochemistry and forced-swim test	↑ in hippocampal neurogenesis in escitalopram, escitalopram + wheel running, and wheel running only, reduced depressive-like symptoms in wheel runners and escitalopram + wheel running groups
[497]	SwHi rats and SwLo rats	6–14	Voluntary wheel running	3	Forced-swim test	Reduced depressive-like symptoms in SwLo wheel runners with concomitant ↑ galanin mRNA in locus coeruleus
[508]	Sprague-Dawley rats	“Adult” status, but actual age not specified	Voluntary wheel running wheel during CORT administration; voluntary wheel running wheel prior to CORT administration; voluntary wheel running prior to and concurrent with CORT administration	2	Forced-swim test, sucrose- preference test, BrdU immunohistochemistry, synaptophysin, and BDNF	Reduced depressive-like symptoms in animals that ran prior to and concurrent with CORT administration, ↑ neurogenesis and cell survival in animals that ran prior to and concurrent with CORT administration, ↔ BDNF or IGF-1 in animals running prior to and concurrent to CORT administration, ↑ synaptophysin proteins in animals running prior to and concurrent to CORT administration
[131]	Fischer 344 rats exposed to chronic uncontrollable stress	“Adult” status, but actual age not specified	Treadmills, motorized running wheels, or voluntary wheel running	6	Shock-elicited freezing and shuttle-box escape	Both forced and voluntary wheel running reduced fear conditioning and ↓ cognitive impairments
[515]	Fischer 344 rats exposed to chronic uncontrollable stress	“Adult” status, but actual age not specified	Voluntary wheel running	6	Shuttle-box escape	Running reduced fear conditioning and cognitive learning, ↓ 5-HT_2C_R mRNA in basolateral amygdala and dorsal striatum
[495]	C57BL/6 mice exposed to chronic social defeat stress	10	Voluntary wheel running	<1	Social-interaction test, open-field test, monoamines	Reduced social avoidance in those that ran 2 hours after stress, ↔ anxiety levels, ↔ in depressive symptoms
[250]	Myshkin mice (Myk/+)	6–12	Administered melatonin or voluntary wheel running	6	Open-field test, elevated plus-maze, light–dark box, accelerating rotarod, EEG and EMG recordings during sleep, and BDNF protein	Both melatonin and PA reduced manic behaviors, melatonin ↑ sleep duration, ↔ in hippocampal BDNF mRNA following exercise
[516]	C57BL/6 mice exposed to lipopolysacharide	16 and 88	Voluntary wheel running	4 for young mice and 10 for aged mice	Tail-suspension, sucrose preference, TNF-a, IL-1B, IL-6, and IFN-*γ*	↔ in depressive- like behavior in presence of ↑ TNF-a, IL-1B, IL-6, and IFN-*γ*
[511]	Fischer 344 rats exposed to acute uncontrollable stress	“Adult” status, but actual age not specified	Voluntary wheel running	6-7	Serotonin and dopamine in striatum	Running prevented stress-induced elevation of extracellular serotonin and potentiated dopamine concentration in the dorsal striatum
[494]	Outbred Hsd:ICR mice stressed by wheel removal	7–9 wks	Voluntary wheel running	6 days	Forced swim, tail- suspension, and behavioral despair	High CORT males deprived access to running wheel showed ↑ depressive symptoms
[510]	C57BL/6 mice exposed to chronic uncontrollable stress	6	Voluntary wheel running	4	Corticosterone and adrenal weight	More rapid corticosterone response but returned to baseline more quickly in wheel runners, ↑ adrenal weight
[513]	C57BL/6 mice exposed to chronic uncontrollable stress	8	Voluntary wheel running	3–6	Corticosterone, BDNF, neurogenesis, open-field, elevated O-maze, dark- light box, forced swim, and learned helplessness	↑ hippocampal BDNF, ↑ neurogenesis, ↑ cortico-sterone metabolites, ↑ anxiety-like behavior in novel and aversive environments
[512]	Fischer F344 rats exposed to chronic uncontrollable stress	“Adult” status, but actual age not specified	Voluntary wheel running	3 and 6	Shuttle-box escape and conditioned fear	6 wks of running ↑ hippocampal BDNF mRNA and protein following stress
[251]	C57Bl/10 mice	8	Voluntary wheel running, fluoxetine, or combination of fluoxetine and voluntary wheel running	3	BDNF, IGF-1, and BrdU immunohistochemistry	↑ in hippocampal BDNF in fluoxetine group only, and ↑ in neurogenesis with fluoxetine group only
[517]	TrkB^hGFAP^ and TrkB Nestin mice that were conditionally ablated for the gene encoding TrkB, the high affinity receptor for BDNF, in a regional and cell-type-specific manner	Postnatal day 15 and “adult” status specified, but not actual age	Voluntary wheel running	6	Dark light test; open-field test; forced swim test, BrdU incorporating cells	neural Progenitor cell deletion of trkB, both in embryos and in the adult, causes ↓ hippocampal neurogenesis and ↓ behavioral improvements following chronic antidepressant administration or wheel running
[514]	F344 rats exposed to chronic uncontrollable stress	8	Voluntary wheel running prior to stress exposure	6	Sleep and temperature rhythms	↑ entrainment of sleep/wake behavior and ↓ disruption of diurnal rhythms of sleep and temperature following stress

PA and MDs in preclinical studies. BDNF: brain-derived neurotrophic factor; CORT: corticosterone; CUS: chronic unpredictable stress; SP: short photoperiod; STAT3: signal transducer and activator of transcription pathway 3; tx: treatment; VEGF: vascular endothelial growth factor.

## Abbreviations

ACTH: Adrenocorticotropic hormone
BDNF: Brain-derived neurotrophic factor
BP: Bipolar disorder
CRH: Corticotropin-releasing hormone
FNDC5: Fibronectin type III domain-containing protein 5
GABA: Gamma aminobutyric acid
HPA: Hypothalamic-pituitary-adrenal axis
IGF-1: Insulin-like growth factor 1
IL-1: Interleukin-1
IL-6: Interleukin-6
MDD: Major depressive disorder
MDs: Mood disorders
NMDAR: N-Methyl-D-aspartate receptor
PA: Physical activity
PGC-1*α*: Peroxisome proliferator-activated receptor gamma coactivator 1-alpha
RCT: Randomized controlled trial
ROS: Reactive oxygen species
SCN: Suprachiasmatic nucleus
TNF-*α*: Tumor necrosis factor-*α*
VEGF: Vascular endothelial growth factor.
